# Pediatric Ambulatory Blood Pressure Patterns and Cardiovascular Risk

**DOI:** 10.1007/s11906-026-01361-y

**Published:** 2026-01-30

**Authors:** Jordan Sill, Elaine Urbina

**Affiliations:** 1 Heart Institute, Cincinnati Children’s Hospital Medical Center, Cincinnati, OH USA; 2 Department of Pediatrics, University of Cincinnati, Cincinnati, OH USA

**Keywords:** Hypertension, Pediatrics, Ambulatory blood pressure monitoring (ABPM), Target organ damage, Cardiovascular risk

## Abstract

**Purpose of Review:**

Pediatric hypertension is associated with antecedent cardiovascular (CV) risk factors and the subsequent development of early subclinical CV disease. Current guidelines recommend the use of ambulatory blood pressure monitoring (ABPM) to diagnose and classify pediatric hypertension into distinct patterns. In recent years, emerging literature has explored the risk factors linked to these ABPM patterns and the associations of these patterns with target organ damage. This review summarizes studies published in the past five years that examine pediatric ABPM patterns and their relationship to cardiovascular risk.

**Recent Findings:**

ABPM can be used to diagnose hypertension patterns, such as masked hypertension and non-dipping, that can not be identified through office-based measurements alone. This review of recent studies highlights multisystem risk factors that are associated with abnormal ABPM patterns in youth. It also presents growing evidence linking these patterns to target organ damage across pediatric populations with varying cardiovascular risk profiles.

**Summary:**

Ambulatory blood pressure patterns in pediatric patients can be used to stratify cardiovascular risk in youth, before the onset of cardiovascular events.

## Introduction

**Fig. 1:**
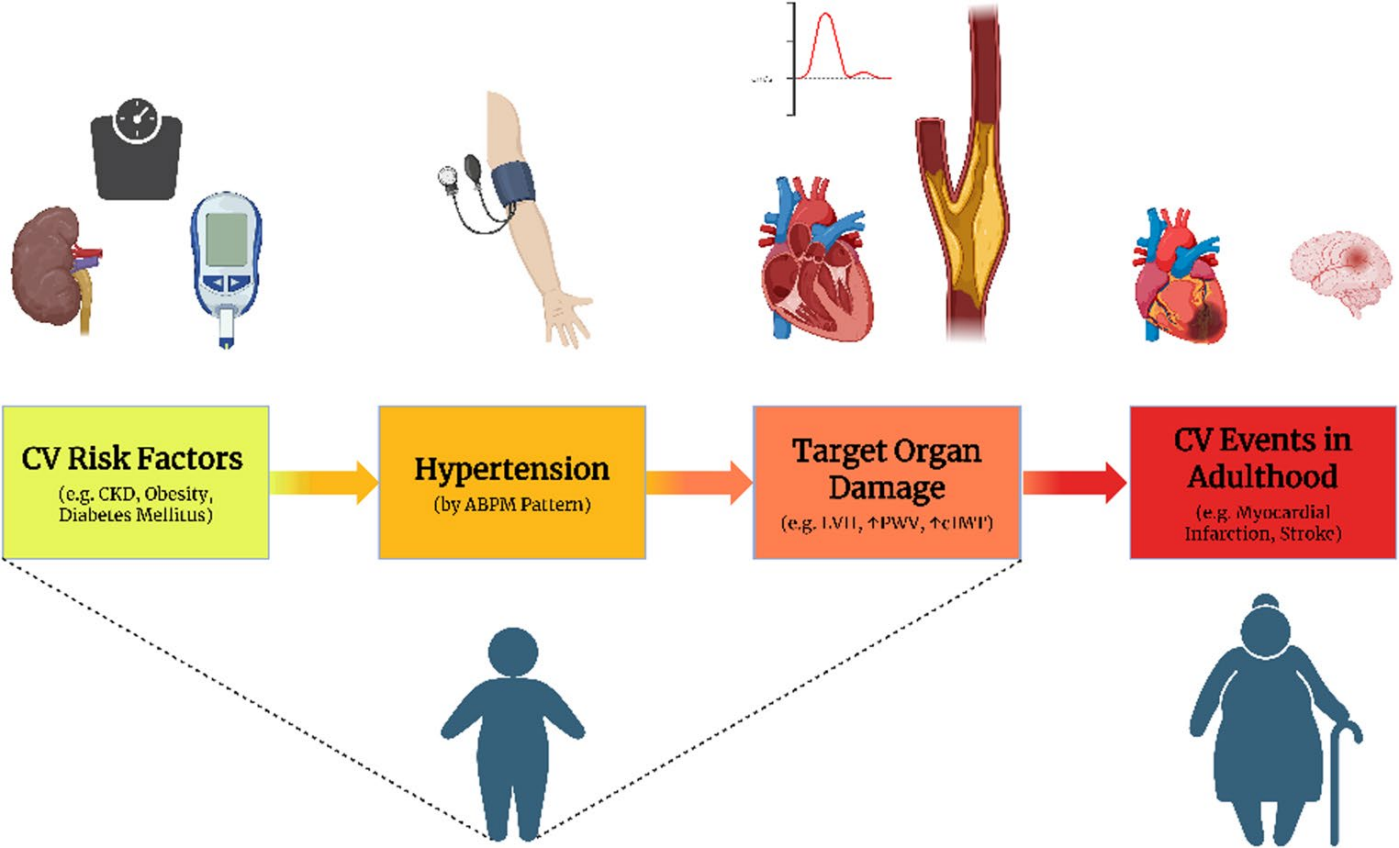
Conceptual framework highlighting the role of hypertension in cardiovascular risk. ABPM identifies distinct blood pressure patterns in youth, linking early-life risk factors to the development of target organ damage. These changes increase the risk of cardiovascular events in adulthood

Cardiovascular (CV) disease remains the leading cause of death with an estimated annual cost of $417.9 billion dollars [1]. Hypertension is a major risk factor for CV Events [1]. Pediatric hypertension affects 2–5% of children [2] and is associated with preceding CV risk factors (Table 1), progression to adult hypertension [3], and early CV events in adulthood [4]. Fortunately, hypertension is a modifiable risk factor of CV events [5]. Because elevated BP is associated with subclinical CV disease in youth, early CV aging can be studied to assess the impact of early-life CV risk exposures because clinical CV disease takes years to manifest (Fig. 1) [6].

**Table 1:** Risk factors associated with hypertension by ABPM Pattern*

Risk Factors	Ambulatory Hypertension	Masked Hypertension	White Coat Hypertension	Nocturnal Hypertension	Non-Dipping Pattern
*Renal*					
Chronic Kidney Disease (CKD)	✓ [7–10]	✓ [8, 10–15]	✓ [8]	✓ [8–10, 16, 17]	✓ [18]
CKD with obstructive sleep apnea	✓ [14]				
*Cardiac*					
Coarctation of the aorta	✓ [19]	✓ [19, 20]			
*Transplantation*					
Heart transplantation		✓ [15]		✓ [13]	
Solid organ transplant		✓ [13]			
*Endocrine*					
Type 1 diabetes mellitus	✓ [21]	✓ [15, 21, 22]			✓ [23]
Type 2 diabetes mellitus		✓ [15]		✓ [24]	
*Respiratory*					
Congenital central hypoventilation syndrome	✓ [25]				
Sleep disordered breathing					✓ [26]
*Genetic*					
Tuberous sclerosis	✓ [27]	✓ [27]	✓ [27]		
Turner syndrome		✓ [28]			
Behçet’s disease	✓ [29]				✓ [29]
Familial mediterranean fever requiring dual therapy					✓ [30]
*Autoimmune*					
Lupus					✓ [31]
*Hematologic*					
Sickle cell disease		✓ [13]			

*Risk factors listed represent conditions that have been associated with the specified ABPM pattern in pediatric and young adult populations based on a narrative review of the literature published within the past 5 years. Associations are not mutually exclusive, and individual studies may report overlap across ABPM patterns

**Fig. 2:**
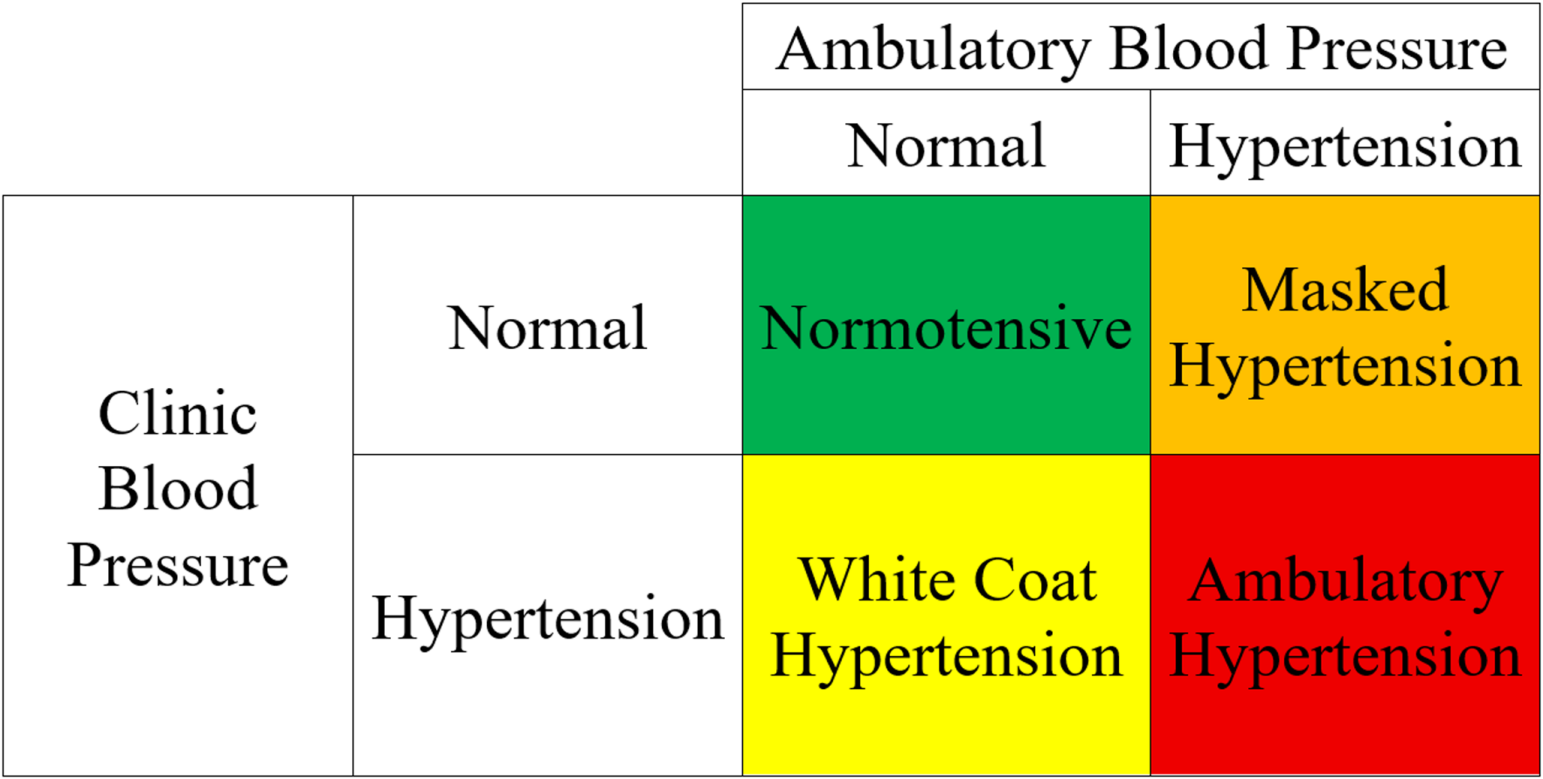
Classification of blood pressure status based on clinic and ambulatory measurements [33]

Ambulatory blood pressure monitoring (ABPM) is the gold standard for diagnosing pediatric hypertension [32, 33]. When combined with office blood pressure measurement, ABPM enables classification into distinct ambulatory blood pressure patterns for better risk prediction: normotension (NT), white coat hypertension (WCH), masked hypertension (MH), and ambulatory hypertension (AH) [33] (Fig. 2). Although clinic hypertension is a risk factor, AH is demonstrated to be a better factor in predicting TOD [34]. ABPM also allows for assessment of nocturnal dipping status, calculated as the percentage decline in blood pressure from wakefulness to sleep [35]. A decline of less than 10% is considered abnormal and has been associated with increased CV event risk in adults [36]. Measures of blood pressure variability may also provide additional risk stratification [37, 38].

While the deleterious effects of elevated blood pressure in childhood on target organ damage are well established, gaps remain in our understanding of the antecedents and consequences of specific ABPM patterns in pediatric populations [33]. This review aims to summarize recent literature on the risk factors for ambulatory blood pressure patterns and the associations of those patterns with markers of subclinical CV disease in pediatric patients.

## Markers of Subclinical Cardiovascular Disease Outcomes by ABPM Pattern

Hypertension in childhood is linked to cardiovascular (CV) events in adulthood [4, 39]. Blood pressure related target-organ damage (TOD), including increased pulse wave velocity (PWV), carotid intima–media thickness (cIMT), and left ventricular mass index (LVMI), predict cardiovascular events in adults [40]. TOD can be detected before clinical events and is associated with childhood hypertension [41–44]. Because CV events seldom occur in youth, studying TOD offers a more feasible approach to risk stratification. Studies have linked pediatric hypertension to TOD in youth [45, 46], but how different ABPM patterns relate to subclinical organ damage in this population remains poorly described. This review adds recent findings on the associations of ABPM patterns with TOD in youth.

### Cardiac Structure: Left Ventricular Mass/Hypertrophy, Left Atrial Enlargement

In children, left ventricular hypertrophy (LVH) is defined by elevated LVMI [47], with published normative data available [48]. LVH can develop as a maladaptive response to chronic, increased afterload [49]. It can lead to systolic ventricular dysfunction and cardiovascular events [40]. Cross-sectional studies have linked hypertension with increased LVMI/LVH [50, 51]. Here we will review recent literature on ABPM patterns on LVH or increased LVMI.

**Fig. 3:**
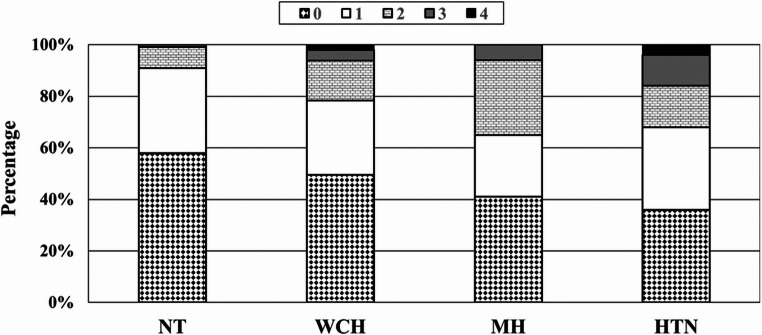
Percentage of participants with target organ damage (TOD) according to ambulatory blood pressure (ABP) phenotype and the number of TOD markers (0–4). Categories include normotension (NT), white-coat hypertension (WCH), masked hypertension (MH), and sustained hypertension (HTN). A stepwise increase in the prevalence of TOI was observed across ABP phenotypes, with significantly higher rates in all hypertensive groups compared to NT participants (*P* < 0.001). Reproduced from Hamdani et al., 2025, with permission [54]

In a study of 352 adolescents with office hypertension and 64 NT controls, participants with AH or WCH on ABPM were more likely to have LVH than NT peers [52]. In a multicenter study of the effect of elevated blood pressure on TOD (the SHIP-AHOY cohort), AH and MH groups exhibited a higher prevalence of LVH compared to WCH and NT groups (Flynn 2025). A meta-analysis of 136 pediatric studies found that MH is associated with increased odds of LVH and higher LVMI compared to NT [13]. Further highlighting the risks of MH is a case study of a single patient who presented to a Pediatric Cardiology clinic with LVH and t-wave inversions that was attributed to MH [53]. The SHIP-AHOY cohort also showed that each hypertensive subtype is linked to a greater number of TOD markers, including elevated LVMI [54] (Fig. 3).

Obesity is an important factor in the development of LVH. In 158 hypertensive children, obese participants had similar SBP z-scores but higher odds of LVH than non-obese peers [55]. In a cross-sectional study of obese Caucasian children at a single Italian center, LVH prevalence was high overall (22.2%) but did not differ between AH and NT groups [56].

In otherwise healthy youth with primary hypertension, a non-dipping pattern on ABPM was shown to be associated with LVH [57, 58]. Another study, however, found no association between dipping status and LVMI in newly diagnosed hypertensive youth [59]. Latency bias may account for this discrepancy. Using cardiac MRI, Sarnecki et al. demonstrated that isolated systolic hypertension is linked to greater LVMI than WCH [60]. Patients with isolated nocturnal hypertension (thought to be a significant contributor to MH [16]) and isolated daytime hypertension had no difference in their LVMI, and both groups had greater LVMI than patients with NT [61]. There was also no difference seen in LVMI between patients with isolated systolic hypertension and isolated diastolic hypertension [62].

Recent studies have applied the ABPM patterns to populations with secondary hypertension. A single center study showed that 6/25 adolescents with repaired coarctation of the aorta had LVH including 2/14 with MH and 1/4 with AH [20]. Patients with AH had greater LVMI but this did not reach statistical significance [20], likely related to the small sample size.

In a single cross section of children with CKD, the odds of having LVH were not different between those with MH and NT although the overall prevalence was high (27.5%) [8]. In patients who have undergone kidney transplantation, patients with uncontrolled hypertension had greater LVMI than NT patients [63]. In the ESCORT trial, 5/19 patients had LVH after kidney transplant but after initiating the treatment arm to maintain ABPM MAP < 75 percentile all 5 patients had regression of their LVMI to the normal range [64].

### Cardiac Function

The association between hypertension and systolic and diastolic dysfunction is well established [65]. This dysfunction is thought to result from hypertension-associated LVH, which leads to progressive stiffening and dilation of the left ventricle over time [66]. Cardiac dysfunction from hypertension also occurs in children [67, 68].

The SHIP-AHOY cohort studied associations between ABPM patterns and cardiac function [69]. MH was associated with systolic dysfunction as exhibited by lower ejection fraction (EF) and higher peak longitudinal strain than other ABPM patterns (Flynn 2025). Lower diastolic function was evident with elevated E/e′ (ratio of early mitral inflow velocity to early diastolic mitral annular velocity, an estimate of LV filling pressure) in MH and AH versus WCH and NT and e′/a′ (ratio of early to late diastolic mitral annular velocities, reflecting diastolic relaxation) was reduced in WCH compared to other patterns (Flynn 2025). No differences in E/A ratio (ratio of early to late mitral inflow velocities, commonly used to assess diastolic function) were found. (Flynn 2025). Vortex formation time (VFT), a novel measure of diastolic function that reflects efficiency of LV filling, was lower in youth with AH compared to NT controls [70]. No other measures of systolic or diastolic dysfunction (e.g. E/A, E/e’, ejection fraction) were different between the groups other than left atrial volume [70], which in adults has been linked to diastolic dysfunction [71]. In SHIP-AHOY, patients with AH had significantly greater odds of having abnormal left atrial volume or left atrial volume indexed to body surface area than MH, WCH, or NT [72].

In patients with CKD, daytime and nighttime diastolic hypertension correlated with reduced left ventricular global longitudinal strain (LV GLS) [73]. Although a greater proportion of patients with AH had abnormal LV GLS, statistical significance was not reported [73]. AH following kidney transplant was associated with lower LV GLS compared to NT, but no differences in EF, tricuspid annular plane systolic excursion (TAPSE), E/A, deceleration time, E/e′, or right ventricular GLS [63].

### Carotid Intima-Media Thickness (cIMT)

Common cIMT measured by ultrasound reveals early atherosclerotic changes [74] related to CV risk factors. Risk factor related elevation in cIMT [75] predict future cardiovascular events [76].

In children and adolescents with AH exhibit greater cIMT than those with NT [6]. In a meta-analysis of patients with MH and TOD [13], one study found a no significant difference in cIMT in patients with MH and NT [77]. In a study of early vascular aging, the actual cIMT observed in youth with primary, AH was greater than chronologically expected [52].

Recent studies have demonstrated associations with cIMT and ABPM in patients with secondary hypertension. In children with repaired coarctation of the aorta and a right arm to leg blood pressure gradient < 20 mmHg, the mean cIMT standard deviation score was found to be 3.1 +/- 1.5 [19]. Elevated cIMT was also associated with LVH and a history of recoarctation [19]. Children with T1DM and MH, AH, or non-dipping showed a significant association between BMI z-score and greater cIMT [21]. The relationship between BMI z-score and cIMT was not significant in those with T1DM and NT [21]. BMI predicted elevated cIMT in regression models in subjects with primary AH [6]. Non-dipping was also shown to be associated with greater cIMT in children with Lupus [31].

Patients with primary snoring were found to have greater cIMT and odds of non-dipping pattern on ABPM, although direct associations between cIMT and non-dipping were not tested [26]. In another group of children with primary snoring prior to adenotonsillectomy, they showed no difference in mean SBP, mean DBP, mean MAP or dipping compared to healthy controls yet had greater cIMT measures [78]. Six months after the surgery, mean MAP and cIMT measures decreased significantly and were no longer different from controls [78].

### Arterial Stiffness

High arterial stiffness is associated with hypertension [79] and with subsequent CV events in adult studies [80]. In youth, a higher burden of CV risk factors over time is associated with arterial stiffness as measured by carotid-femoral pulse wave velocity (PWV) [81], the standard for measuring arterial stiffness [82].

Abnormal ambulatory blood pressure patterns have been associated with measures of arterial stiffness in adolescence. Youth with AH demonstrated PWV values exceeding those expected for their chronological age [52]. In a multi-center study of youth with normal, elevated blood pressure, and hypertension in clinic (*n* = 373), youth with AH, MH, and WCH groups had significantly greater PWV than NT peers [69]. In a smaller cohort (*n* = 85), AH and MH had significantly greater PWV than WCH and NT groups [83]. Meta-analyses have shown that NT youth have significantly lower PWV compared to those with MH [13] and AH [6]. Adolescents with isolated systolic hypertension (ISH), the most prevalent phenotype of primary hypertension in this age group, had greater proximal and thoracic aortic PWV measured by MRI compared to those with WCH [60]. Among youth with type 1 diabetes mellitus, those with AH or MH had greater PWV than those with WCH or NT status [84]. Few data are available evaluating changes in arterial stiffness after treatment but in newly diagnosed essential hypertensives, PWV z-score improved after six months of antihypertensive treatment, but there was no change in LVMI [85].

A higher augmentation index values, a measure of wave reflections related to arterial stiffness, has been found youth with AH or a non-dipping blood pattern compared to NT peers [59]. Central blood pressure, which can be obtained when measuring augmentation index, may more accurately reflect hemodynamic stress on target organs. Central BP has been shown to correlate more strongly with LVMI, carotid intima-media thickness (cIMT), and PWV than brachial systolic blood pressure in adults [83]. Ambulatory central blood pressure [86] was more strongly associated with LVMI and cIMT, but less strongly associated with PWV, compared to peripheral systolic blood pressure [87]. In youth with primary hypertension, central blood pressure and augmentation index were significant independent predictors of the discrepancy between actual cIMT and values expected for chronological age [52]. Augmentation index was higher in patients with essential hypertension compared to NT peers, and in those with a non-dipping pattern compared to those with a dipping pattern [59].

### Other: Flow Mediated Dilation, Cognitive Function, Retinopathy, Renal Disease

Flow mediated dilation (FMD), a measure of endothelial function, has been evaluated in relation to ABPM patterns. Diastolic non-dipping was associated with reduced FMD in patients with lupus [31]. Additionally, children with primary snoring were more likely to have a non-dipping pattern on ABPM and lower FMD than matched controls without snoring, although a direct association between non-dipping and reduced FMD was not evaluated [26].

Two studies have demonstrated associations between ABPM-derived blood pressure metrics and poorer cognitive function in children; however, these studies did not specifically evaluate ABPM patterns [88, 89]. In a single-center study, approximately 10% of patients with office blood pressure > 95th percentile had retinopathy; however, the prevalence did not differ significantly among those with WCH, pre-hypertension, or AH [90].

In a cohort of adolescents with normal kidney function, urine albumin-to-creatinine ratio did not differ significantly across ABPM patterns [69]. Among patients with CKD, those with both daytime and nighttime AH (but not isolated daytime or nighttime hypertension), were more likely to progress to renal transplant compared to NT peers [9]. Youth with type 2 diabetes and daytime hypertension, with or without nighttime hypertension, had greater odds of having albuminuria, an early marker of diabetic kidney disease [24]. In type 1 diabetes, albuminuria is associated with elevated nighttime diastolic BP [84, 91]) and non-dipping pattern [84].

## Persistence of ABPM Patterns Over time

Although tracking of casual blood pressure over time has been demonstrated in multiple cohorts [3, 42, 92], little is known about the persistence of ABPM patterns over time in children. In one study with a median follow-up of 20 months, 92% of NT children remained normotensive, while fewer than 20% of those with AH, WCH, or MH retained their original classification [93]. The Pediatric Nephrology Research Consortium found that 61% of patients under 18 years of age with WCH developed an abnormal ABPM pattern after a median of 14 months follow up, including 23% developing AH [94]. In a study of children with spurious hypertension (isolated systolic hypertension with normal central blood pressure), 23% of participants progressed to AH after one year of follow up [95]. Data from long term follow up is lacking. However, a large cross sectional study in Italy showed a decline in NT prevalence and an increase in AH and MH across decades of life [96], suggesting that these patterns may exist in individuals as well.

## Conclusions

ABPM offers insights into risk stratification in pediatric hypertension beyond office-based measurements, enabling identification of patterns such as MH, WCH, and non-dipping status. This review highlights that literature supporting these patterns as important predictors of TOD, including LVH, arterial stiffness, and increased cIMT. ABPM patterns are associated with a wide range of primary and secondary risk factors, spanning congenital, metabolic, environmental, and lifestyle domains. However, longitudinal data on the persistence of these patterns and their progression into adulthood remain limited.

Despite growing evidence linking ABPM patterns to early target organ damage, implementation of ABPM in routine pediatric care remains underutilized [97]. Further research is needed to improve the understanding of the long-term trajectory of these patterns, their associations with CV events, and to determine the impact of interventions on TOD as well as long term risk. Additionally, studies should explore how integrating ABPM, central blood pressures, and TOD markers in clinical workflows may improve early detection and guide treatment decisions. Ultimately, advancing the use of ABPM in pediatrics may offer a pathway to improve prevention of atherosclerotic cardiovascular disease.

## Data Availability

No datasets were generated or analysed during the current study.
